# New Players for Advanced Prostate Cancer and the Rationalisation of Insulin-Sensitising Medication

**DOI:** 10.1155/2013/834684

**Published:** 2013-03-19

**Authors:** Jennifer H. Gunter, Phoebe L. Sarkar, Amy A. Lubik, Colleen C. Nelson

**Affiliations:** ^1^Australian Prostate Cancer Research Centre-Queensland, Institute of Health and Biomedical Innovation, Queensland University of Technology, Translational Research Institute, Princess Alexandra Hospital, 199 Ipswich Road, Brisbane, QLD 4102, Australia; ^2^Vancouver Prostate Centre, Department of Urologic Sciences, University of British Columbia, Vancouver, BC, Canada V6H3Z6

## Abstract

Obesity and type 2 diabetes are recognised risk factors for the development of some cancers and, increasingly, predict more aggressive disease, treatment failure, and cancer-specific mortality. Many factors may contribute to this clinical observation. Hyperinsulinaemia, dyslipidaemia, hypoxia, ER stress, and inflammation associated with expanded adipose tissue are thought to be among the main culprits driving malignant growth and cancer advancement. This observation has led to the proposal of the potential utility of “old players” for the treatment of type 2 diabetes and metabolic syndrome as new cancer adjuvant therapeutics. Androgen-regulated pathways drive proliferation, differentiation, and survival of benign and malignant prostate tissue. Androgen deprivation therapy (ADT) exploits this dependence to systemically treat advanced prostate cancer resulting in anticancer response and improvement of cancer symptoms. However, the initial therapeutic response from ADT eventually progresses to castrate resistant prostate cancer (CRPC) which is currently incurable. ADT rapidly induces hyperinsulinaemia which is associated with more rapid treatment failure. We discuss current observations of cancer in the context of obesity, diabetes, and insulin-lowering medication. We provide an update on current treatments for advanced prostate cancer and discuss whether metabolic dysfunction, developed during ADT, provides a unique therapeutic window for rapid translation of insulin-sensitising medication as combination therapy with antiandrogen targeting agents for the management of advanced prostate cancer.

## 1. Obesity, Type 2 Diabetes and Prostate Cancer 

### 1.1. Obesity and Cancer Risk

Worldwide rates of obesity have doubled in a generation with a global estimate of ~500 million obese adults (with an additional 1.5 *billion* overweight) being followed by a generation of 40 million overweight children [1]. In both industrialised and developing countries these staggering numbers pose a soaring economic and health care burden as a result of chronic comorbidities including increased rates of cardiovascular disease, hypertension, stroke, and type 2 diabetes (T2DM) [2]. 

Obesity is also a risk factor for a growing number of cancers. Retrospective observational studies and meta-analyses, using body mass index (BMI; mass (kg)/height (m^2^)) as a measure of adiposity, have demonstrated an increased risk of breast, ovarian, colorectal, bladder, kidney, and endometrial cancers with increasing BMI [3–6]. Similarly, obesity attributes a 12% increased risk of prostate cancer diagnosis [3] although studies have varied, with some showing a weak association (relative risk (RR): 1.05 [7]), or a significant risk of high-grade and metastatic cancers, (RR: 1.22–1.55) but not low-grade prostate cancer [8]. These differences may be accounted for by differences in detection bias (using cohorts of closely monitored patients, for example, during diabetes screening), differences in time of  “disease-free” follow-up and patient age [9]. Also, these studies may be limited by the use of BMI as a measurement of adiposity, underscored by the recent finding that specific measurement of visceral fat volume, the more metabolically compromised fat depot in obesity, may provide a much stronger statistical correlate with disease-free survival in cancer patients than BMI [10]. Adding to these statistical differences, there are more practical difficulties of prostate cancer detection in obese subjects. Measurements of prostate specific antigen (PSA), a serum biomarker used for screening, which can also be elevated in nonmalignant disease such as benign prostatic hyperplasia (BPH) or with aging, can be difficult to interpret in obese patients; both lower levels of PSA, due to increased blood volume and increased PSA levels concomitant with increased incidence of BPH [11], have been reported in obese men [12]. Notwithstanding, a recent study by Fowke et al. [9] has found that the association between obesity and prostate cancer persists when these factors are taken into account. 

### 1.2. Obesity and Cancer Progression

What is indisputable from the epidemiology is the impact of obesity on cancer behaviour. Obesity is consistently identified as a significant risk factor for more aggressive disease and an independent predictor of recurrence and cancer-specific mortality for breast [13], endometrial, ovarian [14], and bladder cancer [5] as well as prostate cancer [9, 15]. Men with higher BMI are more likely to be diagnosed with higher-grade cancers and higher Gleason scores and suffer an increased incidence of recurrence [3, 7, 15, 16] and increased cancer-specific mortality than men with a healthy BMI [16–18]. 

The molecular link(s) between obesity and malignancy is the subject of current research and has been recently reviewed [19–21]. Obese adipose tissue creates a hypoxic environment, as well as an overproduction of ROS resulting in oxidative and ER stress [22]. In addition, many bioactive molecules are altered in obesity which could contribute to neoplasia and cancer progression [21] including lipid mediators, inflammatory cytokines, and hormones/growth factors. As adipose tissue expands, a proinflammatory environment develops with increased secretion of cytokines such as IL-6, TNF*α*, and MCP1, from both adipocytes and resident immune cells. These, in turn, act as chemoattractants for further immune cells and thus create a feed-forward loop, perpetuating immune cell infiltration and cytokine production. Cell signalling is impacted, particularly insulin signalling in local and peripheral tissue (e.g., muscle and liver) leading to systemic insulin resistance [23] and sustained elevated circulating insulin levels. 

Dysregulated lipid flux in obesity results in decreased HDL cholesterol and elevated LDL, circulating levels of triglycerides and free fatty acids which have been shown to promote prostate cancer cell survival [24, 25]. Inflammatory lipid mediators such as arachidonate and downstream signalling lipids, such as eicosanoids, prostanoids, and leukotrienes, are also increased [21, 26] and could potentially impact tumour cell biology; arachidonate can also promote steroid hormone production in prostate cancer cells [27]. 

Altered hormonal profiles are also a hallmark of metabolic dysfunction with modulated adipokine production and secretion, including increased leptin, proportional to adipose mass, while adiponectin shows an inverse relationship [28]. Elevated leptin levels have been associated with breast, prostate and colon cancer progression, and leptin promotes *in vitro* cell proliferation and invasion [9, 29], as well as independently modulating inflammation. Reduced adiponectin, which purportedly puts a brake on malignant cell proliferation, has also been observed in a range of cancers [30] including prostate cancer. 

However, it is hyperinsulinaemia, as a result of insulin resistance in classical metabolic tissues, which has been identified as a highly significant risk factor to progression of prostate and other cancers [14]. In prostate cancer, elevated insulin or C-peptide levels (used as a normalised surrogate) have recently been shown to significantly correlate with high-grade prostate cancer and worse patient prognosis [16, 31–36], more significantly than BMI alone, suggesting that at least part of the effect of increased BMI on prostate cancer mortality is related to coincident hyperinsulinaemia [33]. Similarly, a strong association with high C peptide and the development of high-grade prostate cancer of Gleason grade 7 or greater has been identified, independent of BMI [36]. Baseline insulin levels at time of prostate cancer diagnosis have also been shown to be the most significant predictor of lethal prostate cancer, strongly suggesting that insulin is a major factor in prostate cancer progression associated with metabolic dysfunction [35]. Chronically elevated insulin facilitates in tumour tissues increased activation of mitogenic, anabolic, and prosurvival pathways with the increased levels of insulin and concomitant elevated insulin-like growth factor 1 (IGF-1) [37, 38]. We have also recently reported that insulin regulates the expression of novel gene transcripts/splice variants in tumour tissue [39]. In addition, many tumour types, including prostate tumours, upregulate the expression of related insulin receptor (INSR), IGF-1 receptor (IGF-1R), and hybrid INSR/IGF-1Rs [31, 40] further promoting insulin-driven cancer survival pathways [41]. And notably, an increased ratio of INSR/IGF1R expression has recently been described in prostate tumours and adjacent tissue [42], which suggests specifically that insulin signalling plays a key role in these tumours.

Together, the altered hormonal and inflammatory milieu of obesity may contribute significantly to cancer growth and progression via promoting mitogenesis (e.g., leptin, IGF-1, insulin), angiogenesis (e.g., VEGF, IL6, IL8), and invasion (e.g., leptin, IL6, PAI-1, CCL5, CCL2) [19].

### 1.3. Diabetes and Cancer Risk

Obesity is a major risk factor for the development of T2DM, a condition defined by hyperglycaemia in a background of insulin resistance in metabolic organs (e.g., muscle and liver). Diabetes generally develops as pancreatic *β*-cells are unable to respond adequately to increased insulin required to maintain normoglycaemia. Diabetes has been independently associated with increased risk of several cancers including colon, pancreatic, and breast cancer [43, 44]. In contrast, some studies suggest that diabetes may be protective of prostate cancer risk [45–47], perhaps due to the typically decreased levels of androgens in these men; prostate tumours are fuelled by androgens for growth and survival, and testosterone levels are typically lower in men with diabetes [46–48]. However, this is also confounded by a lack of separation of men taking diabetic control agents, such as metformin [46, 49]. Although diabetes is associated with a lower incidence of prostate cancer, postdiagnosis, diabetes increases the risk of cancer-specific mortality [50], suggesting that similar risk factors contributing to cancer progression are involved in prostate cancer as other tumour types. Large cohort studies of diabetic patients are partly compromised by the fact that many studies reporting the relative risk of cancer with diabetic status have not separately defined the proportion of type 1 and type 2 diabetes within these studies, nor analysed the cohorts individually or based on medications [46, 49, 51]. While highlighting the association between hyperglycaemia *per se* and prostate cancer, metabolic risk factors associated with T2DM with potential links to cancer, such as hyperinsulinaemia and inflammation, are likely to lose statistical impact by combining diabetic subtypes; the rate of obesity in T2DM is 52% (86% overweight), compared to 16% in type I diabetes (55.3% overweight), and, as such, the metabolic contribution to malignancy, described above, would be expected to have significantly different impact [52]. Furthermore, type 1 diabetes is characterised by a *loss* of insulin and use of exogenous insulin analogues whereas T2DM is treated with a range of medications including metformin, sulphonylureas, and insulin which vary in cancer risk profiles [53–55], some conferring protection, (metformin) and others increasing risk (sulphonylureas and insulin analogues) [45, 53]. Indeed, this observation has led many to speculate on the potential utility of diabetes treatments as adjuvant cancer therapeutics. 

## 2. Emergence of Insulin-Sensitising Drugs in Cancer 

The observation of increased cancer risk with obesity and diabetes has led to the somewhat more hopeful speculation of potential therapeutic benefit of insulin-sensitizing drugs with the major clinical outcome of lowering systemic insulin levels. Studies have shown some cancer survival benefit in type 2 diabetes patients treated with the biguanide and metformin compared to those treated with insulin or sulphonylureas (stimulate insulin secretion from pancreatic *β*-cells) [53, 56–58]. In addition, the thiazolidinedione class of PPAR agonists [59], the lipase inhibitor, orlistat, and cholesterol-lowering statin class of drugs [60, 61] have also been shown to associate with lower cancer risk. A growing body of *in vitro* evidence is beginning to provide mechanistic detail to these observations. 

### 2.1. Metformin

Metformin, which works in part by activating AMP-activated protein kinase (AMPK), is used clinically in obese and diabetic patients to normalise circulating insulin levels primarily via reduced hepatic glucose output and improve insulin/receptor interactions. Recent clinical studies have suggested that metformin may improve patient outcomes in prostate and other cancers [33, 53, 62–64]. Amongst diabetic populations, metformin has the lowest cancer mortality profile when compared to sulphonylurea or insulin treatment [53, 56–58, 63], in a dose and duration of treatment-dependent manner. 

Apart from normalising systemic insulin, the intracellular effects of metformin in tumour cells have been the subject of recent study and review [41]. Metformin can potentiate several pathways to prevent tumour cell growth and invasion, primarily via blocking anabolic pathways such as lipognesis and protein translation, preventing mitosis and increasing sensitivity to chemotherapy. 

The activation of AMPK in cancer cells blocks cell proliferation by negatively regulating mammalian target of rapamycin (mTOR) control of protein synthesis [43, 65–67]. Selective suppression of translation of mRNAs-encoding cell cycle regulators within the mTOR/EIF4E pathway by metformin has been recently described [68], and the expression of many genes involved in mitosis, such as tubulins, histones, and kinesins is decreased by metformin [69]. AMPK acts as an intracellular energy sensor, responding to fluctuations in the ratio of AMP and ATP. In times of energy deficit (high AMP:ATP ratio), the activation of AMPK blocks anabolic pathways and promotes the generation of ATP (e.g., oxidative phosphorylation, *β*-oxidation). Thus, metformin effectively blocks lipogenesis by inhibiting activation or expression of lipid biosynthesis enzymes such as acetyl Co-A carboxylase (ACC) and fatty acid synthase (FASN), key regulators of the metabolic reprogramming identified in prostate cancer [70]. Likewise, the enzyme responsible for steroid (estrogen) production, aromatase, is decreased in breast adipose tissue with metformin use [71]. 

Another proposed mechanism of metformin is preventing pathways which lead to invasion and metastasis evidenced by the demonstration of metformin to prevent invasiveness in cancer models [72, 73]. Similarly, it has been reported in pancreatic cancer cells that metformin reversed cell invasion by facilitating the reexpression of critical RNA species required for phenotypic maintenance [74]. Based on these results, targeting AMP-activated protein kinase (AMPK) has been proposed as a therapeutic strategy in cancer [75, 76]. 

### 2.2. Lipid Modulators: Orlistat and Statins

The pancreatic lipase inhibitor, orlistat, has its primary effect on reducing hydrolysis of triglycerides and preventing the absorption of dietary free fatty acids. Its major clinical benefit in obesity is preventing caloric intake, thus, reducing circulating lipid levels and improving insulin sensitivity [77]. FASN expression is upregulated in a number of cancers including ovarian, breast, and prostate [78–80] so the discovery that orlistat was a novel inhibitor of FASN [81] triggered numerous studies into the efficacy of orlistat as a therapeutic agent for cancer. orlistat has been shown to prevent *in vitro* and *in vivo* growth of the prostate cancer cell line PC3 and to induce apoptosis. [81]. The inhibition of FASN with orlistat also causes cell cycle arrest and induces caspase-mediated apoptosis [82] and has also been shown to increase sensitivity to chemotherapeutic compounds [83, 84]. 

Statins lower cholesterol levels by inhibiting a critical enzyme for cholesterol synthesis, HMGCoA reductase (HMGCR), which is upregulated in CaP and breast cancer tissue [85]. The role of HMGCR in cholesterol synthesis, and a precursor to steroid hormone synthesis, is likely to be an important link in the association between statin use and a lower rate of prostate cancer recurrence [26, 86, 87]. Indeed, it has been shown that statin use inversely correlated with the risk and progression of prostate cancer [88, 89]. In a large-scale study by Loeb et al. men on statins were older and had high BMI at time of radical prostatectomy, but, despite these increased risk factors, the patients on statins had lower PSA, as well as lower Gleason score, lower tumour volumes, smaller surgical margins, and less risk of biological recurrence [88]. Other studies demonstrate a significant decrease in advanced and metastatic disease [88, 90]. 

### 2.3. Thiazolidinediones

Thiazolidinediones (TZDs) are a class of insulin-lowering (insulin-sensitising) drugs which activate the PPAR family of nuclear receptors, with predominant affinity for PPAR*γ*, highly expressed in prostate cells [91]. Several TZDs are used clinically to treat type 2 diabetes, including pioglitazone, troglitazone, rosiglitazone, and ciglitazone. Their use in other diseases with hallmark hyperinsulinaemia (e.g., polycystic ovarian syndrome and some lipodystrophies) is the subject of an ongoing research [92]. A large meta-analysis comparing TZD use and cancer incidence [93] found a decreased risk of colorectal, lung, and breast cancers with TZDs, but specifically, the use of pioglitazone—which extended protection to prostate and renal cancer as well. This finding has also been demonstrated in other studies where TZD use is associated with improved survival of diabetic prostate cancer patients [59]. In prostate cancer cell models, troglitazone reduced *in vitro* and *in vivo* cell growth via ERK-dependent regulation of p21 and cMyc [94] and inducing apoptosis [80]. Prostate-specific knockdown of PPAR*γ* resulted in the dysregulation of cell cycle control and lipid signalling networks [95]. In ovarian models, differential efficacy of ciglitazone and troglitazone (but not rosi- or pioglitazone) was observed with respect to cell cycle arrest, increased caspase activation, and evidence is now mounting that this may occur via PPAR*γ*-independent mechanisms [96, 97]. In prostate cancer cells TZDs induced cell senescence, via PPAR*γ*-dependent (rosiglitazone) and -independent (ciglitazone) mechanisms [98]. Studies in lung cancer cell models have reported TZD treatment works directly on cancer cells to decrease pathways that lead to invasion such as epithelial-to-mesenchymal transition (EMT) via Smad-dependent and -independent mechanisms [99]. Taken together, there is compelling evidence that current antidiabetes treatments offer therapeutic benefit in cancer.

### 2.4. Exogenous Insulin Analogues

The efficacy of insulin-lowering therapies in reducing risk and progression of cancer is highlighted by the converse, that is, increased risk of cancer in diabetic patients treated with insulin analogues. These include fast-acting analogues such as Aspart and Lispro with similar INSR but reduced IGF1R-binding affinities compared to native insulin [100], and Glargine and X10 with increased IGF1R affinity compared to insulin, and sustained binding kinetics [101, 102]. Nevertheless, the clear benefit of good glucose control in diabetes is worth bearing in mind in regard to cancer risk. 

## 3. Prostate Cancer as a Candidate for Insulin-Lowering Therapy

### 3.1. Advanced Prostate Cancer

At current rates, prostate cancer will affect one man in seven and is the leading cause of cancer deaths in men in western countries [103]. For rapidly advancing, localised disease, patients are generally treated with radical prostatectomy or radiation therapy with a high rate of success (>90%) [104]. However, up to 25% of patients will recur, heralded by a biochemical recurrence measured as a rising PSA, and require secondary treatment. Prostate tumours depend on androgen signalling for growth and survival, and this dependence has rationalised the standard treatment for metastatic prostate cancer, androgen-deprivation therapy (ADT), for decades [105, 106]. The most common method of ADT uses luteinising hormone-releasing hormone (LHRH) agonists or antagonists to medically disrupt hypogonadal feedback loop to inhibit testicular testosterone production, or to a lesser degree direct orchiectomy. An initial surge of testosterone in response to LHRH agonists is often blunted with coadministration of androgen receptor (AR) antagonists (e.g., bicalutamide and flutamide). ADT results in anticancer response and prolongs cancer control; however, after a median time of 12 to 33 months, the cancer resurges, with a second PSA rise, despite castrate levels of serum testosterone (<20 ng/dL) [107]. This disease stage is referred to castrate-resistant prostate cancer (CRPC) and traditionally has had limited effective treatment options, finding that effective therapy for CRPC has been the focus of intense and productive research in recent years with a surge of new agents approved and, in development, many of which re-targeting in new ways the androgen axis. 

## 4. Current Therapy for CRPC 

While chemotherapies such as docetaxel [108, 109] and cabazitaxel [110] provide some survival benefit in metastatic CRPC, therapeutic strategies are increasingly focussing on the inhibition of androgen signalling as evidence mounts that CRPC continues to utilize androgens for tumour growth and driving cell survival pathways [111–113]. There are several mechanisms by which prostate tumours may reactivate androgen signalling, including gain-of-function mutations or alternative splicing of the androgen receptor (AR) that broaden its range of ligands to other steroid hormones (e.g., progesterone, estrogen, and cortisol), antiandrogens (e.g., flutamide and bicalutamide) [114–116], or confer ligand-independent activity [117]. The development of a hypersensitive receptor, via overexpression of AR and/or receptor stabilization, provides a second mechanism that results in AR activation even at low levels of androgens [118–121]. A further mechanism by which CRPC maintains AR signalling is by producing its own androgens from both adrenal conversion and intratumoural intracrine steroidogenic pathways. The observation that, despite low serum levels, intraprostatic levels of DHT and testosterone were high in men treated with ADT [122], up to 50% of intraprostatic levels measured in eugonadal men [122–125], led to the finding by us and others that, under androgen deprived conditions, enzymes required for conversion of adrenal androgens and *de novo* intratumoural androgen synthesis are upregulated in prostate cancer cells [113, 126, 127]. Thus, no longer reliant on testicular androgens, AR signalling can resume within the tumour microenvironment, leading to the development of CRPC. These factors combined have rationalised the recent surge in new antiandrogens for the treatment of CRPC, which target the AR and inhibit activity, or block rate-limiting enzymes in *de novo* steroid synthesis. 

### 4.1. New Inhibitors Targeting the AR

A tranche of new-generation antiandrogens have recently emerged from preliminary clinical trials with promising results; MDV3100 (Medivation Inc.), a small molecule AR antagonist, has recently been FDA approved for secondary hormone treatment for CRPC under the trade name of Enzalutamide. MDV3100 binding the AR blocks ligand binding, impairs nuclear translocation of the receptor, and induces a conformational change that prevents AR transcription and cofactor recruitment [128]. MDV3100 has from 5- to 10-fold greater binding affinity for the AR than bicalutamide or Casodex, but, unlike bicalutamide, has no partial agonist activity [128] and, excitingly, may also block constitutively active AR splice variants [129]. A second AR-antagonist, ARN509 (Aragon Pharmaceuticals), similarly binds AR, prevents nuclear translocation and transcription, and, in early clinical trials, has shown promise for efficacy at lower doses than MDV3100 [130]. 

### 4.2. New Inhibitors Targeting Steroid Synthesis

**Figure 1:**
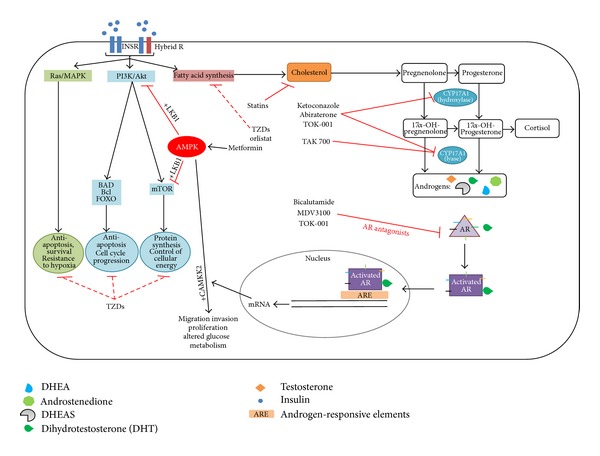
Therapeutic regimens combining insulin-sensitizing drugs and androgen-signalling inhibitors may be beneficial for treating prostate cancer progression following ADT. Androgen signalling is targeted using direct AR inhibitors. Bicalutamide (Casodex) and MDV3100 directly bind AR to prevent activity. Ketoconazole, abiraterone (Zitega), and TOK-001 have dual inhibition of CYP17A1 lyase/hydroxylase activity. In addition TOK-001 directly blocks AR activity. TAK-700 inhibits CYP17A1 lyase activity only and may not require concomitant control of rise in mineralocorticoids as is needed for abiraterone. Hyperinsulinaemia would be expected to increase insulin signalling in prostate cancer cells. Insulin can accelerate *de novo *androgen synthesis [153], which provides the AR with several suitable ligands (Key) and allows AR-mediated transcription of genes needed for CRPC progression. Additionally, insulin signalling may activate several AR-independent pathways (e.g., antiapoptosis and cell proliferation). Insulin-sensitizing drugs, such as metformin, orlistat, thiazolidinediones, and statins effectively block insulin-induced effects such as proliferation, lipid, and cholesterol synthesis and, hence, may be effective for the treatment of prostate cancer. The role of AR on AMPK-mediated cell response is still unclear and may be dependent on availability of cofactors (LKB1 versus CAMKK2).

Antiandrogen therapies are also aimed at inhibiting androgen biosynthesis. Androgens are synthesized from cholesterol via steroidogenesis. Multifunctional enzyme CYP17A1 catalyzes two important steps in the steroidogenesis pathway: the conversion of progestogens to androgen precursors and subsequent conversion to androgens (DHEA and androstenedione). DHEA and androstenedione are then converted to testosterone via 17-*β*-hydroxysteroid dehydrogenase (17BHSD) and then to DHT via RDH5. Currently, both ketoconazole (a pan-CYP inhibitor, Figure 1) and aminoglutethimide (inhibitor of CYP11A1) are used to block key enzymes in the androgen synthesis pathway in combination with ADT. 

Improved, specific CYP17A1 inhibitors such as abiraterone (Janssen) FDA, TAK-700 (Takeda/Millenium Pharmaceuticals), and TOK-001 (Tokai Pharmaceuticals) are now in various stages of clinical assessment for adjuvant use with LHRH agonists. abiraterone, unlike previous CYP17 inhibitors, has a 3-pyridyl substitute and 16,17 double bond, which makes it a highly specific, potent, and irreversible inhibitor of both the hydroxylase and lyase activity of CYP17A1 [131–133]. abiraterone acetate, the oral drug precursor of abiraterone, has been FDA approved with trade named Zytiga for CRPC treatment, in settings of postdocetaxel [134] and now prechemotherapy [135]. TAK-700 is a nonsteroidal imidazole, a potent inhibitor of CYP17A1 lyase activity, with weak inhibition of hydroxylase activity and therefore may not require concomitant control of rise in mineralocorticoids as is needed for abiraterone. TAK-700 is currently undergoing Phase I/II clinical trials. A third molecule, 17-benzoimidazole TOK-001, has combined CYP17/AR inhibitor activity and has shown promising results in preclinical studies including a remarkable decrease in AR protein expression and regression of *in vivo* xenograft tumours [136]. 

While much progress has been made in the emergence of new drugs suppressing AR activity in prostate cancer, the unfortunate reality is that, even in these early trials, patients have become resistant to these new therapies [137]. Both abiraterone and MDV3100, currently the only new-generation drugs with FDA approval, show survival benefits of 4.6 months [134] and 4.8 months [138], respectively, in the postdocetaxel setting. In the prechemotherapy setting, abiraterone treatment offers an increased time to biochemical recurrence of 11.1 months [134, 135]. Early trial results show that MDV3100 has improved efficacy, with an extra 5.3 months to biochemical failure compared to postdocetaxel [138]. The persistent capability of CRPC to become resistant to various means of suppression of AR signalling suggests that a multipronged approach to cancer treatment is required and leads to the research question: what drives prostate cancer aggressiveness and CRPC progression?

## 5. Metabolic Dysfunction Caused by ADT Accelerates CRPC

Metabolic dysfunction is a well-established side effect of ADT [34, 139]. The response to suppression of testosterone in men, irrespective of patient BMI at treatment commencement, includes gain of fat and loss of muscle mass, increased LDL and triglycerides, hypertension, and increased fasting glucose [140]—metabolic symptoms which significantly overlap the comorbidities of obesity. Abrupt withdrawal of androgens by ADT results in hyperinsulinaemia and loss of insulin sensitivity reflected by increased homeostatic model assessment (HOMA) score, within 2 weeks [141], suggesting a direct effect of ADT and is independent of fat mass and age [139, 142]. Major findings from recent studies [36, 143] and our own recent pilot study of men receiving ADT demonstrated a strong trend between elevated C-peptide and more rapid progression to CRPC. This phenomenon appears to be due to a direct inverse relationship that exists between the testosterone and insulin hormonal axes in men [144–149]; testosterone is inversely linked to insulin sensitivity and insulin-sensitising medication increases testosterone [59, 150]; however, the exact mechanisms of crosstalk between these pathways are not well understood [151, 152]. 

Traditionally, insulin has been considered a hormone-controlling metabolic regulation; however, the pathways activated by insulin, including phosphatidylinositol 3-kinase (PI3K)/Akt and Ras/MAPK pathways, have many well-characterised downstream effects relevant to CRPC progression, including the inhibition of apoptosis (e.g., via FOXO and BAD-mediated pathways) [118, 153] and stimulation of cell proliferation (e.g., via mTOR) (Figure 1 *, insulin-signalling pathways*) [40, 154]. Hyperinsulinaemia, secondary to ADT treatment, would be expected to increase the activation of these pathways in the relatively insulin-sensitive prostate tumour. Furthermore, increased insulin signalling would be facilitated by increased INSR expression in advanced prostate cancer [31] (Lubik, Gunter, Nelson, unpublished data). We have recently demonstrated that insulin accelerates intratumoural androgen synthesis [155], a major pathway contributing to progression to CRPC. But, insulin is expected to upregulate a number of pathways leading to ADT failure [156, 157]. Regulation of molecules by signalling downstream of insulin/IGF-1R has also been implicated in CRPC progression, including COX2 [158–160], and nuclear AR chaperone, Hsp27 [161, 162]. Notably, obesity and diabetes may independently regulate these molecules [163–165]. Moreover, serum from obese mice has been shown to induce an invasive phenotype in prostate cancer cell lines [166] suggesting that metabolic changes associated with obesity (including elevated insulin) may drive metastatic transformation. However, chronic insulin signalling in the context of ADT might be complex and highly differentiated from those observed in other cancers [167]. Potential crosstalk between AR and insulin-signalling cascades [168–171] emphasises the potential insulin-driven CRPC progression. And recent studies have implicated the PI3K/Akt pathway activation to inhibit AR signalling [172] resulting in androgen-independent growth. Several other growth factors and cytokines upregulated in obesity, such as IL-6, IL-8, IGF-1, and TGF*β*, may also influence prostate cancer cell proliferation [173–175]. 

## 6. A Future for Combined Therapy

Epidemiological evidence, coupled with the upregulation of insulin-signalling components and insulin-mediated upregulation of CRPC pathways in prostate cancer cells, has collectively rationalised a multidisciplinary approach to treating advanced prostate cancer that incorporates antiandrogen therapy with simultaneous treatment of metabolic side effects induced by ADT. 

Metformin has been the first drug to be explored in this area, with ongoing clinical trials using metformin during active surveillance (NCT01733836) and in combination with ADT (NCT01620593) testing the efficacy of metformin in stabilising/normalising circulating insulin levels. Metformin has a well-characterised safety profile and, while it effectively decreases hyperglycaemia, does not affect blood glucose levels in nondiabetic patients. Metformin may also act directly on tumour cells to reduce cancer cell proliferation via the inhibition of anabolic pathways such as lipogenesis starving the major bioenergetic pathway in prostate cancer cells. However, the ability of the major intracellular target of metformin, AMPK, to potentiate insulin action on cell growth and survival may have more complex regulation in prostate cancer cells via interaction with AR-regulated genes; activated AMPK may potentiate increased prostate cancer cell proliferation and migration when activated downstream of the androgen receptor (AR) [176, 177] under the control of a master regulator calcium/calmodulin-dependent protein kinase kinase 2 (CAMKK2) [177]. AR directly regulates CAMKK2 and is highly expressed in normal prostate with elevated expression in both AR-sensitive and CRPC models of prostate cancer [176, 177]. In studies where metformin activation of AMPK results in cessation of cancer cell growth, signalling is through LKB-1 tumour suppressor [178] suggesting that AMPK is a potentially bifunctional modulator, particularly relevant in the prostate cell, dominated by AR-regulated transcriptional landscape, and conversely may be dysregulated with AR as occurs in prostate cancer. The ability of insulin to accelerate *de novo* steroid synthesis in prostate cancer cells suggests that drugs targeting steroidogenesis in combination with insulin-lowering therapies would be beneficial; trials combining abiraterone and metformin are yet to commence (NCT01677897). 

Atorvastatin (statin) is also currently under examination for treatment in prostate cancer in combination with ADT (NCT01555632). Statins target cholesterol synthesis, the substrate for steroidogenesis and in adrenocortical cells and the ovaries, have been shown to decrease steroidogenic enzymes CYP11A1, HSD3B, and CYP17A1 [179]. Statins were able to slow or halt progression to castrate resistance in LNCaP xenograft models [180] and inhibited prostate cancer growth with greater potency in androgen-deprived condition. The clinical benefit of metformin combined with statin for treatment of biochemical recurrence following primary treatment failure (PSA increase following prostatectomy or radiation therapy) is also under clinical investigation (NCT01561482), targeting lipid/cholesterol metabolism and hyperinsulinaemia in an effort to slow progression. 

The dual activity of orlistat, reducing dietary lipid absorption and as an inhibitor of FASN, could also have an adjunct role to play in mitigating the side effects of ADT which may promote cancer progression. orlistat could potentially be used to reduce systemic dyslipidaemia, normalising free fatty acid and cholesterol levels and thereby reducing substrate for tumour metabolism and biosynthesis. However, dietary fats make up only a fraction of the lipid available to prostate cancer cells, which almost universally upregulate FASN [181]. 

## 7. Conclusion

Prostate cancer is the most common cancer in men [103] and, given its long natural history and with onset beginning in 5-6 decades of life, will continue to rise and plague our ageing population. At the same time we face the growing epidemic of obesity while the risk of aggressive prostate cancer is increased 3-fold with obesity. First-line therapies for localised cancer will fail in nearly 25% of prostate cancer patients, and these men will subsequently be treated with ADT [182]. While effectively treating prostate cancer, ADT induces hyperinsulinaemia [182–185] which independently acts on prostate cancer to upregulate lipid and steroid synthesis and contribute to CRPC progression and which may promote metastases, tumour growth, and treatment resistance. Standard chemotherapeutic agents have had limited benefit in CRPC, and there has been quite promising development of next-generation therapies targeting androgen synthesis and directly AR in combination with ADT. These new targeted agents are generally well-tolerated and significantly improve survival; however, most patients ultimately fail, highlighting the urgent need to understand mechanisms underlying treatment resistance and find rationally informed combined and/or sequential treatment options. An existing toolbox of well-tolerated insulin-lowering therapies has accumulated over several decades to treat type 2 diabetes and metabolic complications associated with obesity. Currently, ADT-induced hyperinsulinaemia is not addressed in prostate cancer patients, despite a significantly increased risk of cardiovascular and cancer-related mortality in these patients [186]; the combination of ongoing research and clinical trials will determine the benefit of adjunct antiinsulin therapy to current standard and emerging prostate cancer treatments.
